# A Critical Review on Wood-Based Polymer Composites: Processing, Properties, and Prospects

**DOI:** 10.3390/polym14030589

**Published:** 2022-01-31

**Authors:** Manickam Ramesh, Lakshminarasimhan Rajeshkumar, Ganesan Sasikala, Devarajan Balaji, Arunachalam Saravanakumar, Venkateswaran Bhuvaneswari, Ramasamy Bhoopathi

**Affiliations:** 1 Department of Mechanical Engineering, KIT-Kalaignarkarunanidhi Institute of Technology, Coimbatore 641402, Tamil Nadu, India; 2 Department of Mechanical Engineering, KPR Institute of Engineering and Technology, Coimbatore 641407, Tamil Nadu, India; lrkln27@gmail.com (L.R.); balaji.ntu@gmail.com (D.B.); bhuvanashankar82@gmail.com (V.B.); 3 Department of Mathematics, SRM Valliammai Engineering College, Kattankulathur, Kanchipuram 603203, Tamil Nadu, India; sasigmath83@gmail.com; 4 Department of Mechanical Engineering, K.S.R.M College of Engineering, Kadapa 516003, Andhra Pradesh, India; skumar3011@gmail.com; 5 Department of Mechanical Engineering, Sri Sairam Engineering College, Chennai 600044, Tamil Nadu, India; bhoopathir.mech@gmail.com

**Keywords:** wood composites, biodegradable polymers, wood flour, wood adhesives, characterization, processing, applications

## Abstract

Waste recycling is one of the key aspects in current day studies to boost the country’s circular economy. Recycling wood from construction and demolished structures and combining it with plastics forms wood-polymer composites (WPC) which have a very wide scope of usage. Such recycled composites have very low environmental impact in terms of abiotic potential, global warming potential, and greenhouse potential. Processing of WPCs can be easily done with predetermined strength values that correspond to its end application. Yet, the usage of conventional polymer composite manufacturing techniques such as injection molding and extrusion has very limited scope. Many rheological characterization techniques are being followed to evaluate the influence of formulation and process parameters over the quality of final WPCs. It will be very much interesting to carry out a review on the material formulation of WPCs and additives used. Manufacturing of wood composites can also be made by using bio-based adhesives such as lignin, tannin, and so on. Nuances in complete replacement of synthetic adhesives as bio-based adhesives are also discussed by various researchers which can be done only by complete understanding of formulating factors of bio-based adhesives. Wood composites play a significant role in many non-structural and structural applications such as construction, floorings, windows, and door panels. The current review focuses on the processing of WPCs along with additives such as wood flour and various properties of WPCs such as mechanical, structural, and morphological properties. Applications of wood-based composites in various sectors such as automotive, marine, defense, and structural applications are also highlighted in this review.

## 1. Introduction

**Figure 1:**
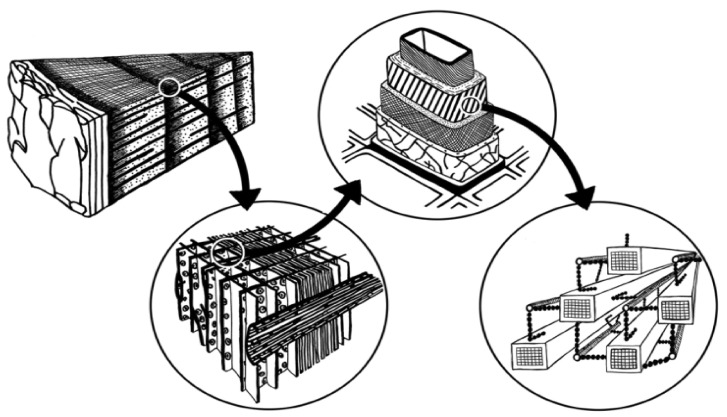
Hierarchical wood structure, Reproduced from [3], Wiley Online Library, 2018.

In contrast to other plants, wood is produced by a perceiving and adapting organism, that is, the wooden body in a living tree is able to carry liquid from the foundations up to the top and to maintain structural performance of the slender branch by absorbing bending stresses caused primarily by breeze [1,2]. This results in the creation of the complicated vertical wood structure, which has an assigned role that is individual to each body. Cell membranes that are compact and the reduction of the bridge cell interior hole area are the elements for attaining structural performance [3]. Figure 1 shows the different zoom elements of the hierarchical wood structure depicting the cell wall arrangement at the nanoscale.

Hardwood composite materials are thermosets that incorporate a small timber in the form of powder or microfibers. They are used in a variety of applications of the dispersed phase [4]. Wood-polymer composites (WPC) are substances or items made up of one or more natural materials or flours but one or a mixture of polymers, such as polyamide, rayon, or latex. Their cheap and superior efficiency, as well as their elevated sustainable development, low moisture absorption, sturdiness against ecological impact such as insects and fungi when compared to timber, high-dimensional data stability over their entire life, and high relative stiffness, have attracted the attention of manufacturers and researchers in recent decades [5]. Natural materials and flours are derived from a variety of sources and contain a variety of polymers. As a replacement for natural wood (for example, in fencing, flooring, and decking), they are especially helpful in wet workplaces or anywhere where wood fibers interacted with liquid: the water-insoluble polypropylene (PP) separates as well as defending the soluble fibers, increasing durability and lowering the need for servicing initiatives, at least to a certain degree [6,7]. Moreover, their applicability in the fields of sound and the motor industry are remarkable. The key benefits of using hardwood as filler are the cost savings and the increase in the environmental properties of the resultant composite that it provides. The first point is self-explanatory, as hardwood particles are often low-cost materials that are commonly derived from farming or commercial leftovers. Furthermore, a significant fraction of quasi substance of coal origin is being replaced by a more environmentally friendly ingredient, often as high as 60 wt. or 70 wt.% of a more eco sustainable element, whereas if monomer is indeed a naturally derived recyclable plastic, the ecological advantage can be enhanced yet further [8,9,10,11].

Fibers as supplements, on the other hand, have some disadvantages. Firstly, there is a restricted selection of matrixes to choose from. Since the natural fiber constituents, lignin and its derivatives (hemicellulose and cellulose), deteriorate in the appearance of oxygen at temperatures of 100, 195, and 250 °C, respectively, it is broadly acknowledged that only plastics with melting points below 195 °C are suitable for use in this implementation [12,13]. In reality, the only applicants are the so-called necessities, which include polyethylene (PE) in both its dense population as well as medium carbon different versions, polycaprolactone (PCL), polyvinyl chloride (plastic), styrofoam (PS), as well as its PCL, including such injection molded plastic as well as acrylonitrile-styrene acrylate (ASA) and PE. As an extra option, some biodegradable plastics, such as polylactic acid (PLA), can be used as matrix materials, and a thermoplastic polyurethane polymer has also been successfully employed as a matrix material. Of course, if cotton or cellulose nanocrystals were utilized in place of natural fibers, a greater variety of polymeric matrix options would be available [14,15,16].

Stiffness and strength are only satisfactory in terms of composite when expensive coupling agents are incorporated into the material formulation; these additives are required for enhancing interoperability between both the wood chips as well as the polyurethane that also might otherwise not communicate whatever pertinent synthetic resemblances [17,18]. This is done in order to create a load-transferring contact among the natural fibers and that is effective. Because WPCs are often brittle, toughening agents including such styrene and butadiene rubber (SBR), ethylene-propylene monomers leather (EPDM), or plastic elastomers are frequently included in their composition [19,20,21,22]. Great affection among the wood fiber and the polymer matrix is crucial to enhance the physical characteristics of wood plastic composites. However, due to the intrinsic characteristics of wood products and the wettability of polymers, the two factors are entirely inconsistent with one another. It is possible to enhance the compatibility of a composite by adjusting the polymer or wood fiber in either a physical or chemical manner, or by applying compatibilizers/coupling substances [23]. Behavior and bonding chemicals have the potential to dramatically increase the efficacy of WPC. Using correctly recruited pairing entities, few authors claim that WPC tensile and flexural strengths can be increased by 2 to 4 times, depending on the test method, stiffness can be increased by up to 40%, impact resistance can be at least doubled, and density and water absorption can be increased by 2 to 4 points of time, relying on the duration of absorption [24].

Because WPCs are naturally composed of a variety of materials, it is possible to speculate that repurposing mixed waste plastics within composites will be easier than reusing them as true raw resources, and that this will result in environmental benefits compared to using traditional woman polymers [25,26]. Use of recyclable plastic in WPCs in manufacturing better recycled goods has also piqued the interest of numerous researchers, who believe that biodegradable polymers have great potential for being used in new products. However, while it has been demonstrated that behavior modification can improve the characteristics of wood plastic composites [27,28], little attention has been paid to analyzing the potential and consequences of several binder. Inside the presence of additive waste plastics, the immiscibility of diverse polymers, along with the inherent compatibility of natural wood and the polymer matrix, creates a challenge in the utilization of mix bioplastics in WPCs. Compatibilizers utilized in WPC, on the other hand, are mainly maleated/maleic grafting backsplash [29,30], which have shown better characteristics in WPCs manufactured from mixes of PE and PP. As previously mentioned, styles can vary; bonded polyolefins can also be employed again for crosslinking of copolymers, which suggests that using terephthalate compounds in WPCs derived from a combination of plastics waste might result in elevated behavior [31,32,33].

Another disadvantage of WPCs is their strong level of combustibility, which must be handled in order to prevent fires. Flame retardants (FRs) are incorporated into WPCs during the blending procedure, and this is the most efficient technique for modifying the flame-resistant qualities of WPCs [34,35]. In the field of flame retardants, ammonium polyphosphate (APP) is a standard, highly effective, and extensively utilized atmosphere flame retardant that has been utilized to improve the fire-retardant qualities of wood plastic composites (WPCs) [36,37]. In terms of improving the fire-resistant effectiveness of APP on WPCs, substances such as elastic graphene sheets, SiO_2_, or CaCO_3_ have been merged with APP to obtain effective fire retardancy for WPCs. Expandable graphite, SiO_2_, or CaCO_3_ were also merged with APP to obtain optimal burn resistance for WPCs [38]. Other inorganic additives such as aluminum powder and magnesium sulphate have been reported to be effective fire retardants for WPCs in addition to APP. Additional organic flame retardants have been developed to enhance the barrier characteristics of WPCs by enhancing the carbonaceous production. A large dosage of mechanical or biological flame retardants described above, on the other hand, is typically required to achieve an acceptable fire-resistance level, as stated previously [39,40,41]. The incompatibility of these flame retardants with the polymer matrices resulted in a decrease for both impact strength hardness, as well as an increase in fracture toughness, in the resulting WPCs. As a result, the technical advances to balance the flame retardancy of WPCs with their other qualities are critical. In prior studies, it has been claimed that the introduction of nano-fillers can enhance the flame retardancy of polymer composites while also improving the mechanical characteristics and heat resistance of those composites at the same time [34,42,43].

There is no doubt that wood-based plastic goods have significant environmental benefits that can be quantified [44]. Furthermore, materials derived from wood waste have the potential to produce low carbon-based products while putting less strain on forests [45]. One option for lowering the environmental impact of plastics and timber debris is to use these wastes to make WPC goods, such as pallets, which are made of wood fiber and polymer. However, in order to evaluate the sustainability impact of WPC pallets, a comprehensive life cycle analysis must be performed. Aside from that, it is crucial to keep in mind that various commodities have varying lifespans, recycling abilities, and recycling potential. It is defined by the International Organization for Standardization (ISO) as being one of the environmental management techniques that addresses the social and environmental risks, ecological consequences throughout a product’s life cycle from raw material obtaining through manufacturing, utilizing edge treatment, recycling, and disposal practices [46,47,48].

In construction components, including flooring planks and screens as well as automotive components, WPCs are extensively employed [49,50,51]. Aside from these, some more particular applications for WPCs have indeed been identified and tried in recent years, decking, cladding, paneling, fencing, and furniture, to name a few examples. For example, WPCs have been used in the manufacturing of pallets [52]. Due to their wide range of applications, WPCs can be considered value-added materials when contrasted with other synthetic structures such as fiber cement composites. Cement-bonded composites, for example, have been employed primarily as building materials in recent decades [53,54]. Construction and demolition wood wastes (CDWs) have demonstrated their potential as a food grain feedstock for the development of WPCs. Mineral wool, in contrast to the hardwood and plastics components of CDW, has been discovered to be a viable primary resource for the production of WPCs [55]. As a result, the manufacturing of WPCs could contribute to the achievement of CDW’s material recovery aim. When CDW is used in the manufacture of WPC, traditional proper disposal operations and techniques such as landfills and incinerators are eliminated. Moreover, the use of CDW as a raw resource for WPC manufacture represents a tangible step towards resource utilization because it eliminates the need for virgin recycled plastic and timber [56,57]

Some other applications of WPC can be found in the construction industry. According to the author’s estimate, terrace boards account for 85% of this in Europe, the rest is mainly processed to façade panels. As these are linear products, they are extruded. WPC decking is mostly made of PP or PE, and in the case of façades the plastic-matrix is mainly PVC-based [58]. The chlorine content gives the compound a fire classification one level better, namely B1 according to German DIN 4102 [59], which is advantageous for façade applications. The modulus of elasticity is also higher, which counteracts the deformation of the material under heat. In recent years, WPC has also become interesting for other industries, namely the packaging and automotive sectors [60]. These are under increasing pressure to minimize the consumption of fossil-plastics. WPC saves petrochemical polymers, and this in the amount of the wood-fibers added. This leads to an almost balanced cradle-to-gate CO_2_-balance. The reason for this is that during their growth, the wood-fibers already absorbed exactly the amount of harmful greenhouse gases from the atmosphere, which is later emitted for production in the form of energy and fuel consumption. WPC is therefore almost climate neutral. The balance is all the more positive the longer the WPC remains in an application [61,62,63].

Building products have a comparatively much longer service life, which in the case of façades is 30 to 50 years [64]. If the material is even recycled at the end of its life, which is already theoretically possible with WPC today but has not yet been implemented industrially, then this has an even more positive effect on the conservation of resources. In addition to technical criteria, sustainability-related aspects could be another reason for building planners to select the innovative WPC material for outdoor purposes, such as the manufacturing of façades [65]. The current review emphasizes the various processing methods of WPCs, properties of WPCs, and wood-based adhesives, their characterization methods, and applications of WPCs. Life cycle assessment and cost analysis of recycled wood composites have also been discussed in this article.

## 2. Processing of WPCs

A thermoplastic or thermoset polymeric matrix does seem to have been reinforced with wood in WPCs which are also two-phase materials. Based on availability as well as desired characteristics of the finished products, the reinforced wood can take any form (fibers, particles, sawdust, or flakes of wood) [66]. In addition to palm leaves and agricultural waste, WPCs can be made from a variety of materials [67]. WPCs might not have been ecologically responsible based on the polymer utilized to combine wood. Due to the lack of widespread use of thermoset polymers in WPCs, thermoplastics and their recyclability seem to be more favorable environmentally [66,68]. The addition of heat was necessary for the wetting of wood of thermoplastic in order to augment the adhesion of the two materials. The melting but rather softening temperature of hard to use plastic should not surpass the decomposition temperature of wood in the thermal blending process. Polyolefins, including polystyrene, higher and lower PE, PP, and polyvinyl chloride, have been the only thermoplastics that meet the above criteria. The compounding process is also challenged by ensuring that polymer melt disperses all through the wood particles in a uniform manner. A product’s appearance and finishing quality are influenced by how well it is mixed. In case of short fiber-reinforced composites, the extent of blending is a critical parameter that can have a negative effect on the mechanical characteristics of the finished products [69,70]. As a result of ineffective or excessive mixing, the wood and plastic fibers are damaged severely. To produce WPCs, thermoplastic methods including single screw extrusion, twin-screw extrusion, injection molding, and so on are the primary methods. In addition to solid or hollow body sections and coverings, films, and foams, these items are also available [71].

First, the components (wood/thermoplastic) are combined, then they are communicated to the second stage, where various methodologies are used to form WPCs in a range of forms other than consistent profiles. Regarding the finished products’ privacy properties, each technique has its own set of privacy parameters. During the production of a one-piece pallet, extrusion has a benefit over injection molding because injection molding burns wood fibers due to excessive heat generated during the high-speed injection. The extrusion process, on the other hand, generates less heat because of its lower shear. Extrusion is well-versed in a diverse variety of die designs when it comes to finished goods shapes [72,73].

The characteristics of the composite materials have been studied in relation to a number of variables, including wood concentrations, coupling agents, as well as impact modifiers [74]. The first step was to parch the WF at a temperature of 50 °C for 12 h, resulting in a water content of 20–30% of the oven-dry particle mass. The WFs were indeed ground in a rotational grinder even without the addition of any extra water after parching. Since being ground to something like a fine powder, the WF was retained in a 60-mesh screen. WF was therefore desiccated in such a furnace at 100 °C with one day to remove the moisture (1–2%) before being used in composite fabrication. After that, a 60-mesh screen was used to collect the fine powdered WFs. Prior to fabrication, the WF was allowed to dry in a furnace at 100 °C for one day to eliminate any remaining water content of 1–2% [75]. Processed in a 30 mm co-rotating twin-screw extruder, the WF, matrix, and coupling agents had a length-to-diameter proportion of 30:1. For each zone, the barrels were kept at 170, 180, 185, or 190 °C. The extruder die was kept at a temperature of 200 °C. After being extruded, the samples were placed in a water bath as well as pelletized. Prior to actual processing, the pellets were deposited inside a closed bag and thereafter left to dry to a water content of 1–2% in an oven. First, from the feed region here to the die zone, samples were heated to 180–200 °C. Process time was about 20 s and pressure was 4–5 MPa with the specimens. As per ASTM D 618 standards, the specimens then seem to have been influenced at 23 °C as well as a humidity level of 50% [76,77].

**Figure 2:**
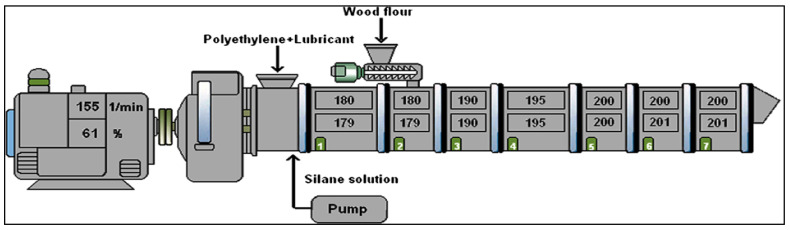
Extruder machine for WPC manufacturing.

WFs were blended with coupling agent and matrix in a high velocity mixer after being oven dried at 80 °C for 24 h. Various quantities of WFs, the required quantity of PP, and the coupling agent were mixed together to prepare the WPCs. Then, the granules of WPCs were again oven-dried before molding at 80 °C for 24 h [78]. For the preparation of WFCs, a twin-screw extruder, as shown in Figure 2, with aspect ratio of 40, screw diameter of 2 mm, a screw speed of 150 rpm, and a material input of 1 kg/h was set and used. A temperature of 180 °C was set between hopper and die as process temperature. Before processing the PP, WF, and fire retardant in an extruder, they were homogenously mixed. As per JIS K7113 standards, an end-gated mold was used in a screw injection-molding machine to prepare the tensile bars which were dumbbell-shaped at a temperature of 210 °C [79].

Five minutes after achieving a constant torque, the inclusion of WF began. Three equal portions of WF were added over the course of two minutes. In intervals of 10 to 25 min, the kneading was kept up until a continuous torque was achieved. After a few minutes, the temperature rose to 150 °C and the combination was left to respond quickly for 20 min. After trying to cool, the WPC was cut into granules with a radius of about 3 mm [80]. The WFC was synthesized in an extruder using a temperature spectrum of 190–230 °C for the compounding process. Injection molding was used to create the compounded samples [81]. Gravimetric-type material feeder-fitted extruders were used to prepare the composites. A twin-screw slide feeder was used to enforce the WF into polymer melt while a peristaltic pump was used to feed the silane solution. In order to prevent the evaporation of water and other reactants in the melt, all atmospheric-pressure ventilations and vacuum ventilations were made impassable. Peroxides disintegrate as radicals when the silane solution passed in through the extruder thus splicing the silane into the composites. Apart from the rate of decomposition of dicumyl-peroxide, the extruder temperature also depended upon the ability to attain sufficient compounding of the material. For a residence time of 55–60 s, the speed of the screw was maintained at 155 rpm and the range of temperature was 180–200 °C. Almost 97% of dicumyl-peroxide was decomposed, which can be approximated theoretically to five half-life times if an actual process temperature for melting was kept around 195 °C. The extruded profile of the WPC was hot-pressed to a thickness of 2.5 mm at 135 °C for 15 s so as to improve the surface texture by straightening it out and was stored either in a thermal bath at 90 °C or at room temperature at 20 °C. The simulated thermal bath, whose relative humidity was almost 100% while that of the ambient atmosphere was 30%, was in the form of a plastic box that contained wires and gratings placed in an oven. The bottom of the thermal bath was filled continuously with water as it gets evaporated often. The composites, immediately after processing, were tested for their ability to cross-link. Cross-linking was prevented by the low temperature in the freezer as hydrolysis was slowed down [82].

### 2.1. Hot-Pressing Technique

The literature on hot-pressing shows that this process is mostly used to produce laboratory samples. Maximum temperatures should not exceed 210 °C and the pressure can be up to 50 tons on specimens with average dimensions of up to 300 mm × 300 mm. With increasing temperature, pressure, and duration, the contact angle increases and water absorption declines. With higher material density, the mechanical strength also increases and the WPC surface appears more uniform. This method is not only suitable for the production of WPC parts from compound granulates but could also be used for the subsequent conditioning of prefabricated WPC sheets. Obviously, the material properties can be further optimized by post-operative hot-pressing under higher pressure, which would have a positive effect on an outdoor application. Some authors reported a separate process of heating up the material in a hot-press and then compressing it in a cold press. In order to use this process for the purpose of industrial “thermoforming” of WPC semi-finishings, several individual processes can obviously be linked together in a similar way. In this respect, this process appears to be superior to the hot-water- and UV-conditioning described above [83].

The hot-pressing method is the heating of WPC-pellets in a metal mold of a certain thickness, which approximately corresponds to the thickness of the sample. Charlet et al. [84] used this method to produce resin-bonded wood-fiber boards of 100 mm × 100 mm × 2 mm, which were produced under 60 kN pressure and 80 °C temperature by 2 h until the resin binder solidified. Ghani and Ahmad [85] used a hot–cold pressing process. They first pulverized WPC compounds made of rice-husks and PE and pressed them into a 14 mm × 14 mm × 3 mm mold at 140 °C and for 14 min to form small test plates, which were then compacted and cooled as “cold press” at room temperature under 1.8 tons of pressure [86]. For hot-pressing, it is important to insert a wax paper between the granules and the hot metal plates. Whether this has a distorting effect on the subsequent surface quality is not known from the literature. Benthien and Thoemen [87] used temperatures of 190 °C to 210 °C for PP-WPC in the pellet press, but temperatures above this level decompose the wood-fiber, which would have a negative effect on the mechanical strength.

### 2.2. Use of Additives

In addition to wood and thermoplastic, WPCs also contain small amounts of coupling agents and lubricants. These additives help the composites work better. By serving a variety of functions, additives seek to improve the final product’s properties. The interfacial bonding among both hydrophobic polymers and hydrophilic woods is critical in the manufacturing of WPCs. Coupling agents because stress is converted here between matrix and fibers at the interface, the composites’ intact mechanical properties are dependent on interfacial bonding. Chemical treatment is one way to improve the bonding; coupling agents such as alkali, acetyl, and silane are commonly used. Fiber treatment with silanes is possible due to the fact that one end of the silane chain reacts with hydrophilic fibers, while the other prefers to respond with hydrophobic polymers, resulting in a chemical bridge between the two ends [88,89]. Hydrolysis of alkoxy clusters on silane to water first produces silanol (Si–OH) groups, which could then respond with hydroxyl groups here on the fiber surface to form hydrogen or covalent bonds. These silanes have been studied the most extensively because of the wide range of applications that they have. Covalent bonds between silane and the matrix have been shown to enhance the hydrophobicity of natural fibers and WPCs’ strength [69]. WPCs are primarily made using lubricants as additives. In order to ensure a uniform movement of the melt through to the equipment as well as a mold during the production of WPCs, the lubricant must be added to the WPC mixture during melting. This is due to the plastic’s high viscosity. Because excess lubrication can lead to a lack of wood/plastic interface linkage, the characteristic is that the amount of lubrication should be kept to a minimum in order to avoid this problem and to select the proper type of handling for wood fiber composites. The oxidized PE lubricant meets the lubricant requirements in many polymer systems [90,91]. Table 1 enlists various lubricants, additives, and coupling agents used during the processing of WPCs.

### 2.3. Wood Composites from Bioadhesives

**Figure 3:**
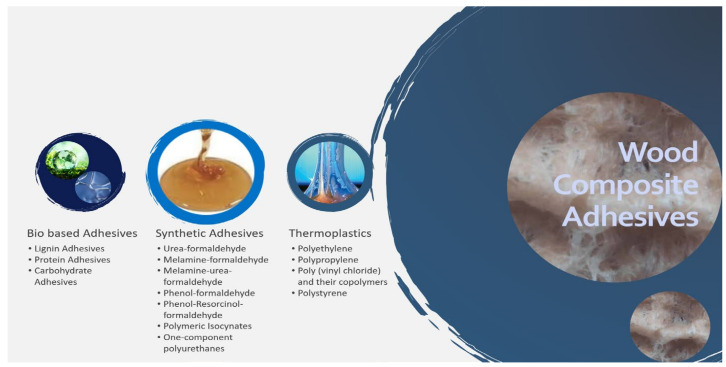
Adhesives for wood composites.

In recent times, an excellent and updated review of wood composites as well as the polymer binders used during their production, and an updated review of wood composites and the polymer binders used in their production was published. Figure 3 shows the review’s focus on the most critical aspects to keep an eye out for when creating high-quality wood composites and the leading binders currently used in the industry. Even though traditional oil-derived adhesives still dominate the wood composite industry for reasons of supply, the review found that progress has been almost unbelievable, and developments dictated by intellectual ferment are caused by a number of external constraints. Formaldehyde and other toxins are being reduced or eliminated by stricter government regulations, consumer awareness, and the resulting drive of the industry to favor more environmentally friendly materials, and eventually, the drive of the industry to decrease or even minimize their own dependence on petrochemicals, owing to the real or envisioned future reduction in oil reserves and the rise in the value of raw materials for purely transgenic products [129,130].

As a wood-based composite adhesive, UF resin has been reactively blended with various starch concentrations, esterified starch, and oxidized starch. UF-starch blends have been found to improve water resistance, formaldehyde emission, and low-brittleness properties [131,132,133]. UF adhesive strength has been found to be comparable to synthetic resin adhesive systems in esterified-starch combined UF and free formaldehyde content was less than 0.3%. The starch adhesive is safe for the human body and could be implemented to wood adhesion. When the UF resin, as well as modified starch, interact, they construct a net structure, which improves the water resistance of starch glue and reduces drying time [134,135]. The formaldehyde emissions can be reduced and the cost was maintained even though starch was used to replace some of the UF in the above systems [136].

Different cross-linkers have been used to enhance the performance characteristics of UF-starch combined glues. The cross-linker isocyanate was used to modify starch adhesives. Carboxymethylcellulose (CMC) and starch adhesive is polyvinyl alcohol, borax, and system for wood composites, isocyanate could be used as a cross-linker, as well as different solid components, isocyanate additions, as well as additives such as PVA and acrylic emulsion have all been researched for their effects on starch adhesive bonding strength as well as water resistance [137,138,139,140]. To increase the starch’s water resistance and bonding strength, additives as well as isocyanates have been added. Cross-linking the cornstarch-UF blend framework with hexamethoxymethylmelamine (HMMM) resulted in an ecologically responsible wood adhesive. Several of the commercially available urea-formaldehyde plywood adhesives for the inside use have mechanical characteristics similar to this one [141,142].

#### 2.3.1. Lignin

Traditionally, the majority of the world’s lignin supply has come from pulping waste. Pulp, as well as paper mills, uses these lignin-derived fragments as boiler fuel because their value is so low. They have a wide range of structural units, from near-native to severely degraded, in their composition. In order to improve the adhesive characteristics of the derived adhesive, modifications as well as cross-linking must be made to the lignin structure [143]. Lignin is made up of C_6_C_3_ phenolic units that are linked together by cross-linking. Chemical organic molecules in lignin include hydroxyl, methoxyl, and carbonyl groups. Lignin chemical groups can be identified using Fourier transform infra-red (FTIR) as well as liquid chromatography, elemental composition, pyrolysis-GC/MS, UV spectroscopy, and wet chemistry methodologies such as methoxyl content analysis as well as nitrobenzene oxidations [144,145,146]. Correlation between the 1H and 13C atoms remaining residues of lignin are very often found to contain cellulose, carbohydrate, and protein contaminants that can be detected using 2D NMR instead of 1D NMR [147,148].

Several studies have examined the use of lignin-based adhesives. Wood particles’ self-bonding properties as well as their improved performance through enzyme treatment have both been extensively studied in the literature [149,150,151]. Kai et al. [152] examined how lignin functionalization could be used to develop sustainable materials such as biopolymers as reinforcement fillers, antioxidants, and UV adsorbents, antimicrobial agents, and carbon precursors as well as materials for tissue engineering and gene therapy. It is reported recently the fundamental understanding of lignin solubilization using SANS and NMR, which will be very useful for analyzing functional lignin for adhesive synthesizing. Zhao et al. [153] recently disclosed similar findings. PF (phenol-formaldehyde) resins, which are used to make plywood, have traditionally included lignin as a partial replacement for the phenol in the resin. Resins with lignin in their chemical structure have lower reactivity, which is an issue when shorter cure times are required. Even though 50% of kraft lignin could provide excellent outcomes in terms of resin viscosity, storage stability, as well as bonding capacity, the pressing time would have to be increased by 30%.

**Figure 4:**
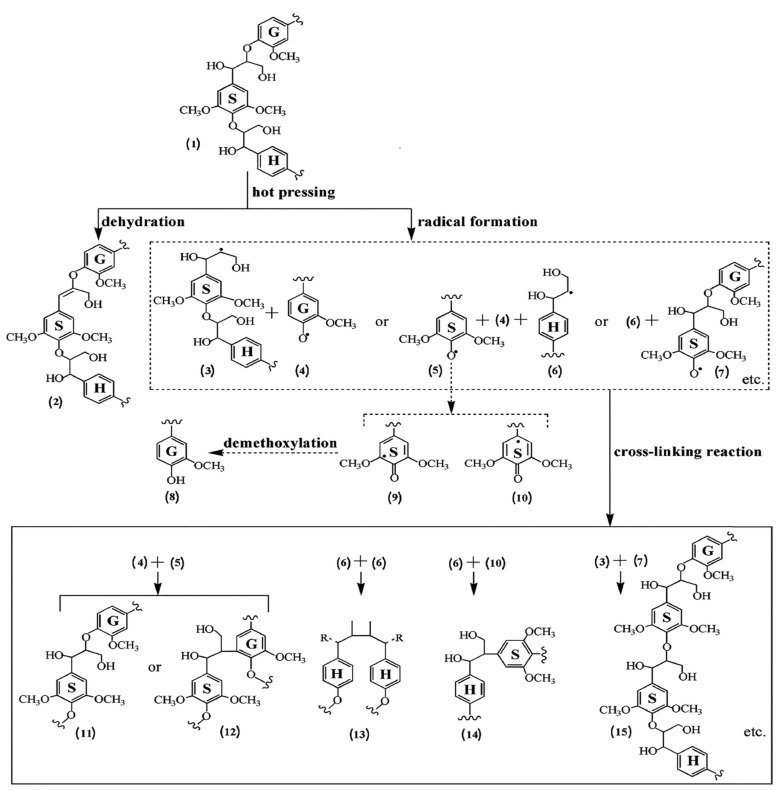
Cross-linking mechanisms for lignin, Reproduced from [157], ACS Publications, 2017.

Lignin derived from those to include more species of wood (bamboo, eucalyptus) has no effect on the bonding properties of plywood when used in commercial mixes, but only 15% of the phenol in commercial mixes can be replaced by lignin. Some researchers have claimed that lignin, glyoxal, as well as pMDI or tannin can completely replace PF resin in fiberboard production [154,155]. MDI isocyanates respond with lignin’s phenolic as well as aliphatic hydroxyl groups to form urethane clusters. Lignosulphonate was combined with glyoxal and pMDI by Mansouri et al. [156]. It is possible to make particleboards with strong internal interfacial adhesion using lignosulphonate-glyoxylate lignin. While glyoxal is not toxic, it is less responsive than formaldehyde and therefore less useful. Lignin cross-linking mechanisms shown in Figure 4 resulted in self-bonding within the molecules.

#### 2.3.2. Tannin

Hydrolysable tannins as well as condensed tannins are two types of natural polyphenols called tannins that are hydrolysable and condensed, respectively [158]. Many plant species contain tannins, and only very few have that much accumulation to make extraction worthwhile. There are a wide variety of plants that contain tannins, including pine, oak, and chestnut, as well as wattle, eucalyptus, myrtle, maple, birch, and willow. The adhesive characteristics of tannin extracts can be affected by different extraction methods. Powdered tannins are the end result of extracting plant material, purifying the isolates, and spray drying it [159]. Pectins, sugars, amino acids, other polymeric carbohydrates, and some other substances are also extracted [160].

In the production of phenol-formaldehyde resin, hydrolysable tannins have really been effectively used more as a substitute material for phenol (up to 50%) [161]. Nevertheless, their low macromolecular structure, low phenol substitution level, limited global production, and relatively high price end up making them less interesting in comparison to compressed tannins [154,162]. Tannin concentrates with a yearly output of more than 200,000 tons account for as much as 90% of the world’s advertising tannin manufacturing [160]. Because of the polyphenol content as well as formaldehyde reaction of these reactive polyphenols, tannins can be used as wood-based panel glue. With formaldehyde as a cross-linker, tannins could be used alone or in a mixture with amino-plastic and phenolic resins to form adhesives. For interior grade MDF, tannins can be used to replace some or all of the phenol in phenol-urea-formaldehyde resins as well as generate 100% tannin resins [163,164].

Diverse tannin sources and timing of tannic inclusion in the production chain of MDF manufacture (for example, during deliberation of wood shavings) have been proven to have an impact on the features of resultant panels [131]. Gonultas et al. [165] and Ucar et al. [166] created and evaluated glue compositions for wooden purposes that were made with a variety of binder and tannin granules from Turkish red pine bark. By analyzing the formaldehyde interaction including both concentrated and hydrolyzed tannin, Ozacar et al. [167] discovered a thermoplastic tannin-based hardwood glue solution that may be used to bond wood. Some research has concentrated on generating resins that are completely clear of formaldehyde by mixing tannins with the other bio-based materials, such as protein, for instance [168]. With the development of particleboard adhesive that is based on tannins, which are extracted from industrial lignocellulosic wastes such as chestnut shell and chestnut bur, as well as eucalyptus bark and leaves, Santos and colleagues [169] explored the feasibility of simply eliminating formaldehyde from adhesive formulations. Nath et al. [170] investigated the qualities and prospects for the manufacturing of particleboard using a tannin-based adhesive derived from mangrove species, and their findings were published in Nature Communications. Cui et al. [171] used cellulose nanofibers in tannin-based glue for plywood manufacture, and they found a significant effect on the mechanical qualities of the panels generated as a result of their experiment.

Formaldehyde emissions from tannin adhesives are quite low due to the phenolic composition of the adhesive. The uses of non-emitting hardeners, as well as tannins which are healed by auto-condensation in the lack of aldehydes, have been shown to further reduce emissions [172]. The autocatalytic hardening of reactive tannins, such as procyanidins, can occur without the use of an external catalyst in the presence of these tannins. It is possible for softer tannins, such as condensed tannins, to undergo auto condensation if a little quantity of alkaline SiO_2_ is present at a high pH, but this is rare. During the process of auto-condensation, the O_1_–C_2_ connection of the phenolic recurrent cycle is broken, which causes auto-condensation among the reactive C_2_ of the free link and free spots in the phenolic unit of some other polymer [154,173]. Auto-condensation may therefore happen at ambient temperature when the pH of the tannin adhesive is elevated, resulting in an increase in the stiffness of the tannin adhesive according to the pH requirements of different tannins; for example, mimosa tannins’ auto-condensation happens at alkaline pH, and *Acacia Nilotica* spp. tannins’ auto-condensation occurs at their initial acidic pH [174].

#### 2.3.3. Starch

Starch is one among the naturally occurring polymeric materials and has gained research focus in various applications such as additives, food packaging, adhesives, and papermaking industries owing to its advantageous features such as cheap cost, better adhesive nature, abundant availability, and renewability [175,176,177,178]. Starch is made of two different polysaccharides such as amylopectin and amylose while these two polysaccharides are constructed with glucose of various sizes and shapes. Properties of the wood adhesives are governed by the ratio of amylopectin and amylose present in the starch which in turn depends on the biological origin of the starch [176]. Properties of starch-based wood adhesives were influenced by hydrogen bonding and these bonds were relatively weaker when compared with the chemical bonds. Poor resistance towards water absorption is also due to the easy bond formation of hydrogen bonds with water molecules. Hence, modification of starch for improving its resistance towards water is important so that it can be widely used in adhesive-based applications [179]. It was stated in many research works that the wear resistance and bond strength of starch-based wood adhesives improved when they were combined with compounds like formaldehyde, tannin, polyvinyl alcohol and isocyanates.

Few studies focused on preparing and grafting starch-based wood adhesives with vinyl acetate while ammonium persulfate was used as an initiator [141,180]. It was stated in the study that graft efficiency played a significant role and influenced the bond performance of starch-based wood adhesive [181]. Esterification of starch is the simple and conventional chemical modification which converts the hydroxyl group of the starch into an ester group so that the hydrophobic nature of the starch was enhanced. Some authors prepared an esterified corn starch by initializing a reaction using maleic anhydride and cross-linking the same using polyisocyanate pre-polymer. It was noticed from the results that the optimal pre-polymer proportion was 10% by weight which resulted in 12 and 5 MPa of dry and wet shear strengths, respectively [182]. Other researchers prepared the starch-based wood adhesive using an auxiliary agent along with blocked isocyanate. A few researchers synthesized eco-friendly starch-based bioadhesive for wood panel manufacturing through the grafting of vinyl acetate in corn starch and making the compound to undergo cross-linking polymerization reaction with methylol acrylamide. Hydrophobicity of the above obtained adhesive was measured to be more than 1 MPa which was attributed to the complex network structure formation and enhanced density of the density due to cross-linking [177,183].

In some other studies, the reaction of urea-formaldehyde resin with various proportions of bio-based starch, oxidized and esterified starch to formulate wood and wood-composite adhesives. It was noticed from the tests that the blending of starch with urea-formaldehyde systems rendered lesser formaldehyde emissions and low adhesive brittleness [184,185]. Meanwhile, blending urea-formaldehyde with oxidized starch rendered an adhesive with resistance towards high temperature, oil, chemical, and aging, and better insulation properties [186]. A few researchers tried to enhance the properties of the starch-based adhesives by adding suitable additives or fillers into them. In some of the experiments, silica nanoparticles were used as fillers in starch-based adhesive to enhance the bonding strength, hydrophobicity, and thermal stability. It was noted from the results that addition of 10% by weight of silica nanoparticles resulted in shear strength values of 2.98 and 5.12 MPa in wet and dry conditions, respectively. From all the above discussions, it could be seen that starch-based adhesives can be a potential bio-based adhesive for the processing of WPCs. Table 2 lists various types of bio-based adhesives used for the manufacturing of WPCs along with their processing parameters and strength values [187,188].

#### 2.3.4. Soy Protein-Based Adhesives

Soy protein has been used as an adhesive since the early period but its utilization as a bio-based adhesive began only during late 1930s. After being commercialized, soy protein adhesives were used as adhesives for paper and wood and in paints and coatings, it was used as a binder. Caustic treatment was used for the denaturation of soy-protein adhesive when it was used as an adhesive for manufacturing plywood. However, the main disadvantages when they were caustic-treated include less solid content, higher hydrophilicity, low biological stability, and smaller pot lives which restricted their usage only in interior applications [189]. In the early 1970s, all the soy-protein based adhesives were replaced by a mix of soy-protein adhesives with synthetic adhesives such as urea-formaldehyde and phenol-formaldehyde. These adhesives exhibited better resistance towards moisture and higher strength but they were less biodegradable.

Usually, it was seen that soy-protein adhesives were relatively cheap with lower pressing temperature and ease of handling. They developed a bond among the WPCs with relatively higher moisture content. It was also noticed that soy protein adhesives had lesser pot lives and high viscosities, thus resulting in the formation of WPCs with lesser resistance towards water, low strength, and lower biological degradation [190]. Soy protein adhesives were also sensitive towards changes in pressing conditions, pH, temperature, and ionic strength while the properties of the adhesives were greatly governed by the content of protein present in it [191]. Viscosity of the soy protein adhesives were reduced by protein hydrolysis or by the usage of lower content of solids during its processing. Hydrolysis results in disintegration of macromolecules of protein into small fragments, thus ending up in lower bond strengths and this treatment can be carried out either by enzymatic reaction or hot caustic treatments [192]. Usage of soy protein adhesives to fabricate particle boards from straws instead of fabricating them from woods was the least studied in much of the research. Since straw surface contains wax and silica in considerable quantities, they are more hydrophobic and hence soy protein adhesives suit better to them when compared with formaldehyde-based adhesives [193].

**Table 2:** Processing parameters of bioadhesives for WPC manufacturing.

Sample No.	Wood Panel Material	Main Adhesive Element	Time of Pressing (min)	Pressing Temperature (°C)	Density (kg/m^3^)	Bending Strength (MPa)	Shear Strength (MPa)	Ref.
1.	Particle board	Lignin	8	200	710	0.38	-	[194]
2.	Particle wood	Corn starch	-	-	-	-	7.5	[195]
3.	Particle wood	Corn starch	1640	30	-	0.35	-	[196]
4.	Particle wood	Kraft lignin/Soy protein	10	180	-	-	6.5	[197]
5.	High density fiberboard	Corn residue and cationic starch	8	235	1130	0.45	-	[198]
6.	Particle board	Pine-based tannin	8	200	-	0.99	-	[199]
7.	Particle board	Maritime pine-based tannin	8	230	670–690	0.46–0.52	-	[200]
8.	Particle wood	Modified soy protein and Sorghum lignin	11	212	-	-	6.22	[201]
9.	Particle wood	Modified soy protein and Extruded sorghum lignin	11	212	-	-	5.78	[201]
10.	High density fiberboard	Lignin from softwood kraft	17	180	1345	0.67	-	[202]
11.	Particle board	Mimosa tannin and pine-based tannin	8	200	717	0.36	-	[203]
12.	Particle board	Tannic acid powder	21	210	1060	0.51	-	[204]
13.	Particle wood	Commercially condensed tannin	7	150	471	-	0.99	[205]
14.	Particle board	Citric acid sucrose	11	210	910	0.38	-	[206]
15.	Medium density fiber board	Modified mimosa tannin	6.5–8.5	165–190	750–850	0.7	-	[207]
16.	Particle board	Organosolved lignin	5	228	720	0.81	-	[208]
17.	Particle wood	Water washed cottonseed meal	22	110	-	-	4.56	[209]

**Figure 5:**
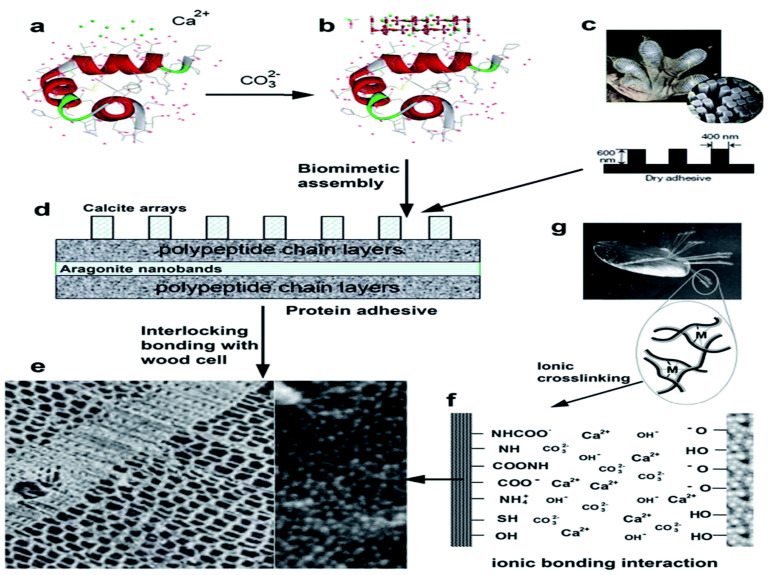
(**a**,**b**,**d**) Synthesis of soy protein adhesives, (**c**,**g**) gecko and mussel adhesive proteins, (**e**,**f**) mechanical and ionic crosslinking mechanisms at the surface, Reproduced from [211], Royal Society of Chemistry, 2017.

Polyamides are the most frequently used agent for inducing cross-linking reaction in soy protein adhesives. Yet, due to the availability of low solid content and high viscosity in polyamides, alternative agents for curing were also addressed in various studies [191,193]. Some studies reported the usage of a new curing agent obtained from ammonium hydroxide in water and epichlorohydrin while manufacturing plywood panels from soy flour. The hydrophobic nature of the adhesive was enhanced by the incorporation of NaOH during the adhesive formation. It was noted from the results that ionic bonding was formed from the modified soy protein adhesive when the adhesion process happened in the WPCs at higher pH values [197,210,211]. Figure 5 shows the schematic of cross-linking mechanisms of soy protein adhesives adopted from natural mussel and gecko-based adhesives.

### 2.4. Post-Treatment of Wood Composites

In order to understand the application-based development of WPCs, it is very much essential to know about the various post-treatment processes involved in treating WPCs. Two predominant methods prevail: post-treatment using ultraviolet (UV) radiation and using boiling water. UV-lamps are also used to prove the aging resistance of WPC in climatic chambers. The research literature offers comparatively more studies on this subject, and they report a significant color change as fading and brightening of the material with a correspondingly long exposure time [212,213]. Chaochanchaikul et al. [214] derived a decreasing hydrophobicity of the WPC-surface from reduced contact angles after 720 h of artificial weathering under UV-lamps with 313 nm wavelength. This is at first in contradiction to Khan et al. [215] which, however, shows that an initial hydrophobicity of the plastic-surface is reversed with prolonged exposure, which can be explained by increasing photo-oxidation and roughness. Peng et al. [216] reported obvious micro-cracks on the surface in this context.

The research on UV-irradiated WPC shows that the material not only heats up as a result of the irradiation energy, but also has positive effects on essential mechanical material-properties, which comes from the cross-linking of polymer chains. This would even increase the performance of WPC in outdoor applications, at least during their early life-phase. More importantly, however, it is possible to reach temperatures at which plastic deformation is feasible under UV-exposure with correspondingly high dosage and exposure-time. In the research literature on UV-conditioned WPC to date, however, this has never been a central question, which is why more precise information on surface and material temperature as a function of intensity and duration is lacking. Investigations on this subject with the aim of a “post-heating” process would thus complement the existing literature. However, material heating by means of UV-lamps takes time and can have a damaging effect if carried out too intensively [217,218].

The current research literature on the boiling test in connection with WPC samples consistently reports maximum heating temperatures of 100 °C under atmospheric pressure and maximum duration of 2 h. This results in significantly higher water absorption and swelling of the material due to the hygric properties of the wood-fibers. There is also a destruction of the outer polymer layer, which favors the absorption of water by the fibers behind. How other mechanical and physical material properties react to hot water immersion is hardly reported [219]. At least in the case of roughness, a significant increase can be assumed. Due to the limited maximum temperature of 100 °C, or 120 °C under overpressure, sculptural forming of WPC appears to be more difficult. The small amount of information from the literature would justify further studies on the physical and time-dependent material behavior due to boiling water storage, but the long conditioning time does not seem very practical for the purpose of industrial thermoforming [220].

Conditioning WPC in boiling water is explicitly mentioned in the material testing standard DIN EN 15534-1 [221] and serves to prove the surface quality, especially for coating with synthetic resin-paints or bonding with veneers. The maximum temperature boiling water can reach is 100 °C, which initially limits the heating of WPC. Cooking under higher pressure, as usually used in vapor pressure-boilers when preparing food, generates temperatures of up to 120 °C. This would already allow plastic deformation of WPC. However, the research literature hardly reports about this conditioning process. Alnajjar et al. [222] carried out the boiling test on WPC-60Wood/40PE. The authors demonstrated 30% to 50% more water absorption of the WPC-samples by boiling for 2 h than by water immersion below room-temperature. Similar results are reported by Mohamed et al. [223] on WPC-70Spruce/30PE. The reasons for the increased water absorption were investigated in more detail by Li et al. [224] on WPC-50 Bamboo/50PE. The authors found that boiling damages the fiber-matrix bond, which promotes water absorption in deeper fibers and makes the surface rougher.

## 3. Properties of WPCs

An injection molding process and extrusion were used to prepare the WFCs. Investigation of mechanical and thermos-dynamic properties was carried out with WF loading as a function. Characterization techniques such as scanning electron microscopy (SEM) and differential scanning calorimetry (DSC) were used to interrelate the morphology of the fiber/matrix interface with the effects of fiber-silane thermo-chemical vapor deposition treatment for WF and a maleic anhydride (MA) graft co-polymerization technique. Theoretical models for the stiffness of the composites such as the Halpin–Tsai/Tsai–Pagano micro-mechanical model were used to compare the results in order to investigate the MA enforcement into fiber/matrix interface and the induced phenomenological effects of silane coupling agent [225].

### 3.1. Mechanical Properties

A possible solution to enhance the mechanical properties of natural fiber-reinforced composites, suggested by several researchers, is the hybridization of fibers with inorganic fillers. The mechanical properties of WPCs may not be significantly affected by fiber type, but it has also been reported that the type of fiber and the lignin, cellulose, and hemicelluloses content have a strong influence on mechanical properties. Addition of WF enhanced the mechanical properties of composites, but simultaneously it increased the burning speed of the materials [226,227,228].

#### 3.1.1. Tensile and Flexural Properties

The mechanical properties such as tensile strength, elongation at break, toughness, fracture energy, and Young’s modulus of the WPCs was measured with an Instron tester (Model: 4201), Zwick/Rowell model Z010, universal testing machine (UTM) following ASTM D638, ISO 527-2 standards with the speed of cross-head as 10 mm/min [229,230,231]. The standard dimension is 20 mm length, 12.5 mm width, and 3 mm thickness [230]. The impact strength of the specimen was tested by using an Impact tester (Model: TMI 43-01) following ASTM D 256 standards. According to ASTM D 790 and ASTM D 7264 standards, the flexural tests were carried out at room temperature with a load capacity of 10 kN in Zwick/Rowell model Z010 UTM [229,231]. According to ASTM D 790-86 standard, a specimen of dimension 70 × 12 × 3 mm^3^ were prepared for a three-point bending test and compression test conducted at room temperature in UTM with a cross head speed of 1 mm/min and 0.5 mm/min respectively [229]. A cross-head speed of 2 mm/min, support span of 140 mm, and a square plate specimen of dimensions 200 mm × 30 mm × 10 mm were the requirements and parameters for a four-point flexural test. For evaluating Young’s modulus through rate of strain, strain gauges were used. A constant temperature of 23 ± 1 °C and a relative humidity of 50 ± 5% were considered while taking all mechanical measurements [230,232].

The tensile strength, tensile modulus, and strain at break of the composites were analyzed. The product of cross-head speed and time gave displacement which was used to calculate the rate of strain [233]. All the prepared specimens had an approximate aspect ratio of 2 and were square in shape. Molybdenum sulfide wax was used to lubricate the precisely machined parallel faces of the specimen. The graph depicted a huge fall of strength and stiffness with respect to time for all compositions of WF. The interfacial adhesion between the fiber and matrix was majorly reduced due to the hydrophilic nature of the composites. This indirectly affects the stress transfer between the fiber and the matrix due to poor interfacial bonding. When a newer bond forms between cellulose and water molecules, the intra-molecular hydrogen bonding was diluted [82,234].

#### 3.1.2. Impact Strength 

How well a façade material absorbs impact loads from hail and storms can also be expressed by its impact strength according to DIN EN ISO 179-1 [235]. It describes the amount of impact energy in Joule, which the material can absorb before it breaks. The impact strength test is performed in the literature on WPC as the Charpy impact test on unnotched specimens. In this test, an oscillating hammer pendulum hits the sample with a certain kinetic energy and breaks it around its weak axis, i.e., its thickness. The fracture energy can be determined from Δh, which is the difference in pendulum height before and after the pendulum passage, and from the hammer weight m. Nevertheless, from the research literature, it is evident that the impact strength decreases with increasing fiber-content, but this reduction is assumed to be of a higher level in the case of hardwood [236,237]. With regard to thermoforming of WPC under hot-pressing, the following hypotheses appear interesting for an experimental clarification: H-10: Type-B specimens exhibit higher impact strength due to hardwood-fibers at comparatively lower share. H-11: Hot-pressing increases the impact strength in A- and B-specimens due to higher surface hardness. Confirming the hypotheses would mean that hot-pressing can compensate for the disadvantage in impact strength resulting from the use of cheaper softwood-fiber or higher fiber-content [238].

#### 3.1.3. Surface Hardness

The surface hardness of a façade material is also a criterion of usability. This is because permanent defects can become visible on the building skin as cavities, especially due to mechanical influences, e.g., branches falling in storms. Similarly, shock-like impact of hard objects can cause cracks or even fractures in the façade. The Brinell hardness is described by DIN EN 1534 [239] using a Lloyd’s test machine. Mostly, a 10 mm carbide ball and 300 kg force over 10 s on 50 mm × 50 mm samples is applied. After pressing the carbide ball into the surface of the material, the Brinell hardness is calculated from the measured force and the surface of penetration. The current research literature on hot-pressed WPC-specimens reports surface changes only in the context of microscopic examinations and describes the effect of fiber content on density and surface hardness [240]. After intensive literature research, there is no study which directly links the relevant parameters under hot-pressing with those of surface quality. Nevertheless, there are some sources which report WPC-investigations without aspects of temperature-related preconditioning. Hot-pressing could bring about an optimization here. Therefore, the following hypotheses should be tested in connection with surface quality: H-8: Type-A samples have a lower surface-hardness due to higher fiber content and softwood. H-9: Hot-pressing increases the surface-hardness of both types due to higher material density. If the surface strength is increased by hot-pressing, this increase can be used for a higher fiber content, which makes the WPC-compound in the façade more sustainable [238].

### 3.2. Physical Properties

When determining the decisive material properties of WPC for exterior applications, mechanical parameters are initially obvious. Previous research mainly reveals that for WPC, as a wood-based material in façades, mechanical strength is decisive, and in particular the bending and fastening mechanisms. However, these properties are mostly correlated with the hygric properties of WPC [241]. The high bio-part, which in the case of WPC is fixed with the wood fiber content, has a negative effect on the physical properties, and this in turn feeds back into the mechanical resistance-values. Finally, there are also optical criteria, which can be expressed in terms of the material’s tendency to change color and shape, and which in turn are a result of hygric material-behavior. Obviously, physical parameters have a high informative value in material characterization towards “post-heating” WPC for thermoplastic forming. Few prominent physical properties such as water absorption capacity and material density play the main role when it comes to assessing the physical behavior of WPCs [238].

#### 3.2.1. Water Absorption Behavior

Increased water absorption of WPC material can significantly reduce the durability, which makes the façade application inefficient. Whether and how much water a WPC-element absorbs depends on the surface quality. As already shown with contact angle method, the wettability gives an indication of the degree of permeability for moisture. However, investigations into water absorption must also show how quickly water diffuses through the material and when saturation occurs. The method for measuring water absorption is defined by test standard ASTM D 570, whereby water absorption is expressed as the %-actual change in sample-weight over time. Water absorption tests were conducted on the specimen which was oven-dried at 105 °C for 24 h. At a normal temperature of 23 ± 2 °C, the specimen was submerged in water for 2 h, after which they were wiped off with a cloth to remove the superficial water and then weighed. This process was repeated after 2 h again and weighed after 24 h [231,242]. Results of water immersion tests of MAPP and PP WF composites for 2 h and 24 h followed same pattern of increase in water absorption as the content of WF increases due to their hydrophilic nature. However, the results were just the opposite for the wood samples because of its hydrophobic nature whereas the water absorption could be more due to the presence of more spots as wood content increases. Attributes such as the presence of lumens, hydrogen bonding sites, and the interfacial gap between reinforcement and matrix were the main reason for water absorption in WF composites [243].

Immersion tests were conducted on rectangular specimens of dimension 35 mm × 12 mm × 3 mm to determine the moisture content as per DIN 52375, ASTM D 570-81 [230] standards. At least three specimens for each sample were taken for the test and their volume (from the specimen dimensions) and moisture content were determined [185]. Within a time of 24 h, different readings of weight gain were taken from the specimen soaked in distilled water at room temperature before which they were conditioned to reach a constant weight. When the readings reach saturation by giving the same value in three consecutive measurements, the final weight gain was calculated [230]. Different pressures were deliberately generated to vary the density of the pores and to demonstrate effects on hygric material properties. Correlations between water absorption and temperature, pressing force, and duration have not been investigated so far. Nevertheless, it seems plausible that, at least with increasing pressing force, the density will be higher, and the surface will seal itself at higher temperatures by closing cavities. Both should be reflected in reduced water absorption-coefficients. Therefore, the following hypothesis is formulated: H-4: Type-A zero samples show higher water absorption due to the larger fibre-content. H-5: Hot-pressing leads to reduced water absorption for both types due to closed surface pores [238,244].

#### 3.2.2. Density

Density already played a major role in water absorption and seems to correlate negatively with this property. It is also regularly determined during physical tests on WPC-formulations. It was applied in a study by Bekhta et al. [245] as a dependent variable during hot-pressing of solid wood-veneers, and the authors demonstrated an increasing density with rising pressing temperature and force, which had a positive effect on the hydrophobicity of the veneers. Hoong and Paridah [246] achieved with both parameters higher bond-strengths between wood-chips and the resin-matrix in their explanatory model. When the pressing time was increased from 18 min to 20 min, the fracture modulus already grew by 25%. Research on the density of WPC was also conducted by Stark et al. [247] on WPC-50Pine/50PE, and in a similar way as the cited solid wood-veneers of Bekhta et al. [245]. Likewise, to Hoong and Paridah [246], a higher density led to higher strengths with respect to modulus of rupture and the stiffness. There is a significant difference in fiber-content between the sample types. Here, too, it is to be expected that Type-A, with 20% more fibers, will result in a lower density also because softwood is used. Regarding density, the following hypothesis can be formulated for a study: H-6: Type-A samples have a lower density due to the higher fiber content and softwood. H-7: Hot-pressing leads to a higher density for both types [238].

#### 3.2.3. Contact Angle and Color Change Measurements

In the contact angle method, a liquid drop, usually 5 μL to 10 μL of distilled water, is applied to the surface of a solid sample, e.g., WPC. The angle between the liquid and solid surface is the contact angle. The hydrophobic property of the surface can be measured from its size. In the research literature on WPC, the contact angle is used as a measure of the weather resistance of WPC [248]. For angles θ ≥ 90°, the surface is considered non-wettable and water-repellent, which in the case of WPC indicates lower water absorption. Since this may be a temporary phenomenon, angle measurements must be carried out as a function of time. The wettability of the composites increased with increasing WF content. The incorporation of the coupling agent decreased the wettability of the composite specimens compared with untreated ones. The test result showed that WF could be utilized in the production of the filled composites because of the surface properties of the composites [84,249].

The research literature shows that the wettability of WPC increases with higher surface roughness and exposed wood-fibers, i.e., contact angles decrease. This is due to the hydrophilic properties of the wood-fibers and the increasing degradation of the polymer chains when exposed to UV-light under weathering. If hot-pressing now makes the WPC-surface smoother and denser and additionally causes thermal decomposition of wood-fibers close to the surface, as stated by Ayrilmis et al. [86], then the wettability should decrease with increasing temperature, pressing time, and pressure, and contact angles should become larger. This has a positive effect on a façade application with thermoplastic WPCs [250]. The following hypothesis can be formulated: H-2: Type-A samples have a higher wettability due to the larger fiber content. H-3: Hot-pressing leads to higher contact angles and lower wettability for both types [238].

According to relevant research literature, color changes in test specimens after conditioning are measured using the color space system (CIELAB). For this purpose, appropriate spectrophotometers are used, e.g., Minolta CM-2500d [25], StellarNet EPP2000 [212], or Macbeth Colour-Eye 7000 [213]. The CIELAB-method calculates color differences in brightness, which ranges from 0 (black) to 100 (white). The values range between −300 and +300 in each case, meaning the further away the value is from 0, the more intense the color tone. The total effect is expressed by index values. Most reported change-effects describe a fading of WPC test specimens, mostly due to UV-light [251]. In this regard, the characteristic value ΔE stands for the total alteration according to ISO 7724 [238,252].

#### 3.2.4. Interaction Behavior

The properties of WPCs are highly dependent on the interaction between wood and polymer [95]. The interaction behavior of WPCs reinforced with linear LDPE was evaluated by Okasman [253]. To improve the interfacial bonding between the matrix and the wood flour, various compatibilizers were added. He conducted tensile testing, impact testing, and SEM analysis. From the results, he found that the mechanical strengths of the WPCs were improved significantly due to better interfacial bonding between wood flour with the LDPE matrix. The influence of the coupling agent and lubricants on the crystallization at interphase regions of wood/PP composites was studied and it was found that there is no difference in the kinetics of the crystal formation and also lubricants that tended to interfere with wood–PP coupling dispersed throughout the transcrystalline region around the fiber [254]. The enhanced dispersion of wood flour particles in the HDPE matrix, ensures efficient interfacial adhesion between filler and matrix which improve the properties significantly [255].

### 3.3. Thermal Properties

Evaluation of thermal properties of WPCs such as thermo-gravimetric analysis (TGA), differential scanning calorimetry (DSC), and flammability or fire retardancy behavior is currently considered to be important since most WPCs are subjected to outdoor applications and some temperature-influenced environments. The following sections deal with the various studies conducted on WPCs to assess their thermal behavior.

#### 3.3.1. Thermo-Gravimetric Analysis 

TGA was used to investigate the thermal behavior of the WF, matrix, and their resulting composites. The tests were conducted with 15 mg mass of sample under a flowing nitrogen atmosphere with a heating rate of 10 °C/min between 35 °C and 700 °C according to ASTME1131-08standards [256]. TGA/DTA measurements were performed on a Netzsch STA 409 TG analyzer. A sample was cut into small pieces and conditioned in vacuum at room temperature for 24 h. Degradation of WPCs usually occurs at a large temperature spectrum at both nitrogen and oxygen atmospheres. However, in contrast to the polymer matrix, some mass loss of WPCs in nitrogen atmosphere occurs in the temperature ranges 30–160 °C, 170–230 °C, and 410–590 °C while the mass loss of the polymer matrix occurs mainly from 310 °C–490 °C. Wood degradation in the temperature range from 200 to 350 °C is attributed to the disintegration of hemicellulose and cellulose while from 250 to 500 °C, it can be attributed to the disintegration of lignin [4,257]. Wood and polymer matrix degradation overlap between 200 °C and 350 °C which means that step separation for wood and matrix could not be achieved. However, when WF was degraded between 200 °C and 390 °C, then the matrix was degraded between 390 °C and 500 °C, and finally, by changing the inert atmosphere to oxidative atmosphere, the char of WPCs burned residue-free [256,258].

#### 3.3.2. Differential Scanning Calorimetry 

The melting and crystallization behaviors of WPCs were assessed through DSC using a Perkin Elmer, Mettler-Toledo DSC 821 calorimeter apparatus equipped with a cooling attachment, under a nitrogen atmosphere. The composite samples were heated from −50 °C to 185 °C at the rate of 3 °C/min before which 10–15 mg of the samples was preheated to 50 °C for 5 min. Afterwards, the samples were glazed 20 °C/min to room temperature. Two heating steps interspersed with a cooling step from 20 °C to 210 °C at a constant rate of 10 °C/min were carried out. The samples were analyzed in standard aluminum DSC pans [257,259]. Thermal properties of the composites such as fusion/crystallization enthalpies Hc and Hf and melty (Tf) and cristally (Tc) temperatures were examined using a non-isothermal DSC analyzer. Alternative heating and cooling of the samples was done between 210 °C and −60 °C in two stages; heating to 210 °C and cooling them to −60 °C as first stage and heating the samples again from −60 °C to 210 °C as second stage, at a rate of 10 °C per minute [260]. The heating process was performed twice; glass transition temperatures were obtained from the second run. DSC thermo-grams depicted the development of double melting peaks, transference of the melting temperature, and abridged heat of melting depicts the imperfect polymer crystal structure when WF is impregnated into the matrix. During the transition from molten to crystalline state, WF enables the fast formation of transcrystalline structures along the fiber surfaces which portrays WF as good nucleating agents. Due to WF impingement on bulk formed crystals and disordered crystal growth at the interface of matrix/fiber, some discontinuities originate in the matrix crystal structure which led to the reduction in whole polymer crystallinity [225].

#### 3.3.3. Fire Retardancy Behavior

In engineering applications, fire retardancy is an important behavior if WPCs are considered as a material for construction. WPCs result as combustible and fire-prone materials since both wood and polymer materials are inherently combustible in nature. In order to mitigate this issue, enhancement of flame retardancy of WPCs deems prior importance. Usually, resistance towards fire can be achieved by ignition through fire or developing the resistance to the propagation of fire. Fire retardancy in a material can be developed basically by the formation of a barrier within the material [261]. It has been stated in much of the literature that such barriers act as an insulation within the material, thus mitigating the propagation of fire by reducing the rate of heat transfer. The flammability characteristics of the WFCs specify the fire performance of the material that is evaluated and classified during compounding process by flammability test [262]. In order to enable the fire retardancy, formation of phosphate of the material in presence of oxygen is important as most of the constituents in WPCs are considered to be the good sources for oxygen. Effective flame retardants such as chlorine and bromine halogenated compounds may be used but as they give out toxic gases during the reaction, they should be avoided. Studies by Sain et al. [263] showed that due to the addition of fire retardants, only a marginal drop in mechanical properties was found and to bring down the burning rate of WPCs to 50% without usage of flame retardant, 25% magnesium hydroxide was effective. According to Gracia et al. [264], self-extinguish materials were developed from PE-based composites due to the addition of fire retardants when reagents of hydroxide or phosphate were used. Experiments by cone calorimetry conducted by Stark et al. [265] arrived at a result that phosphate or hydroxide and various other fire retardants could be used in WFCs.

The influence of fire retardants on the composite’s flammability was studied by two types of burning tests, namely horizontal and vertical tests. Before the test, the specimen was dried at 80 °C for 24 h. When the sample was positioned horizontally and when one of its ends was exposed to a natural gas fueled flame for 20 s, it is known as the horizontal test. Similarly, when the sample was positioned vertically and when one end of the sample was exposed to the flame for 10 s, then it is known as the vertical test. The height of flame and the angle of flame for horizontal and vertical tests were 10 mm, 30° and 20 mm, 30°, respectively. Burning time, which was measured, indicates the time of the flame to reach the second reference point from the first, which are 80 mm apart. In order to study the effect of dripping of the specimen from the fire source, a wire sheet was held at a distance of 10 mm under the specimen. If the specimen is self-extinguished or incombustible in the horizontal burning test, then it is subjected to the vertical burning test. Class of the sample was determined by the sample dripping, its burning state, and combustion time [266,267]. From all the above discussions, it could be stated that the fire retardancy of WPCs was improved by the addition of materials promoting fire resistance.

## 4. Life Cycle Assessment of WPCs

The ecological implications evaluation of various sources, which include WPCs, has been using the life cycle assessment (LCA) technique to analyze the possible prospective implications or mechanism [49,268]. A review of previous research on WPCs’ LCA can be broken down into two categories: (i) studies trying to compare WPCs to other components (e.g., wood) as well as (ii) studies evaluating the ecological consequences of WPCs produced from various raw resources (original materials versus reprocessed materials). The environmental impact of WPCs produced from original as well as recycled (waste) materials has been studied by Sommerhuber et al. [269]. The primary unprocessed substances were virgin wood as well as waste wood, both of which contained HDPE as well as recycled HDPE. They discovered that WPCs produced from reprocessed as well as waste materials had lower environmental impacts than WPCs produced from virgin resources. WPCs made from virgin wood as well as pure glass fiber or reprocessed mineral wool, as well as WPCs made from virgin wood as well as virgin or recycled PP, were studied by Vantsi and Karki [270]. There were significant reductions in ecological implications of WPCs when recycled mineral wool was used rather than virgin glass fiber. These environmental impacts included global warming, acidification, and eutrophication, as well as abiotic depletion potential. Recycling PP has been found to reduce the risk of global warming and the depletion of abiotic resources. Previous studies on the evaluation of the ecological implications of WPCs, such as the ones cited above, focused on the ecological implications of WPC products rather than the ecological implications of WPC manufacturing as part of a CDW management solution [271].

Consequence LCA, which would be assumed to be critical in determining the impact of utilizing a specific pallet on the overall system has been omitted from previous studies, including those cited above. It is critical to look into the distinctions between attributional as well as consequential LCA findings and conclusions, as well as their applicability in situations involving waste recycling. The assumption that all pallets accomplish equally well throughout their life cycle was made in all previous studies. Pallets created of distinct substances have various life expectancies, repair times, and recycling rates, which were not regarded in any of the studies; from the initial to final stage, LCA incorporates the concept of end-of-life (EoL) care. LCA’s final outcome could be significantly impacted by the EoL allocation’s methodological differences [272,273].

**Figure 6:**
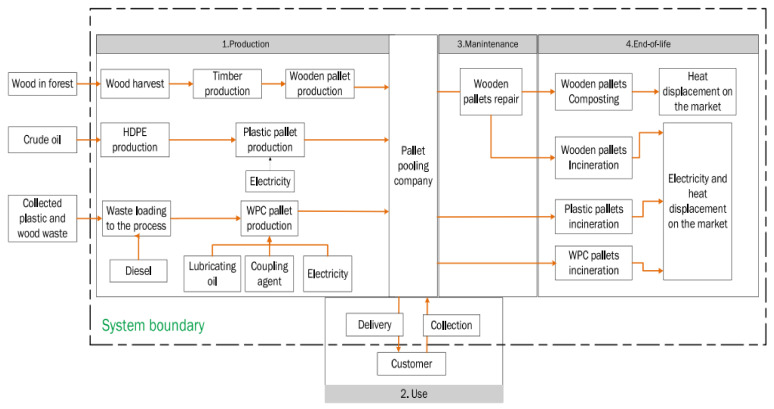
Scope of ALCA studies, Reproduced from [271], Springer, 2021.

**Figure 7:**
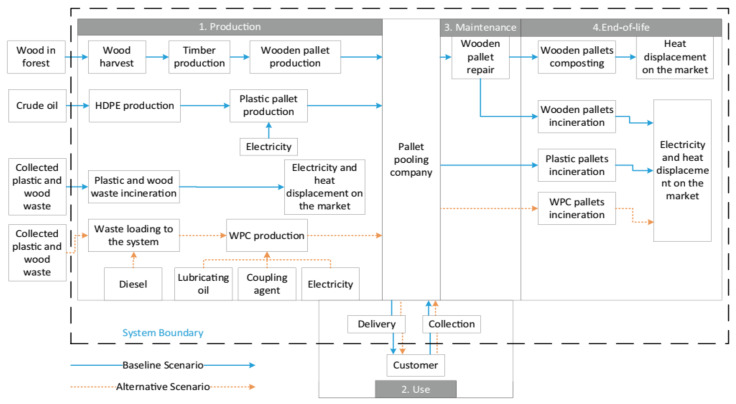
Scope of CLCA studies, Reproduced from [271], Springer, 2021.

Environmental implications of WPCs can be assessed using one of two LCA approaches. An attributional LCA (ALCA) examines the environmental implications of the physical flows that enter and exit the product’s life cycle as well as its subsystems. Consequential LCA (CLCA) examines the production system as well as the systems connected to it that are expected to change during the production, consumption, and recycling of the item. Using the cradle-to-grave approach, the ALCA considers all aspects of a product’s life cycle, from its initial use of raw resources from the ecosystem in the version of elementary flows, such as those generated by nature, to its final disposal, which includes emissions into the atmosphere and water, as well as the creation of waste [274,275]. Figure 6 and Figure 7 shows the scope of ALCA and CLCA studies carried out on WPCs from their cradle to grave.

Certain factors, such as raw material reliability (e.g., possible contaminants), accessibility, the requirement for the generated WPC, utilizing end-of-life stages for WPC-derived goods (e.g., wood and plastic), physical and mechanical characteristics of various WPC types, and improvement of the production methodology, were not taken into consideration in a few LCA analyses carried out. This opens the door to more investigation. It is fascinating to see how WPCs wind down at the end of their useful lives. Only in the manufacturing process can CDW WPCs be recycled [276]. EoL WPC material restoration is restricted because the crops are not yet prevalent. This raises the question as to whether this technique would then only extend the life among these substances with one cycle or if, with an advanced take-back framework, it might offer a technique for progressing toward that circular economy [277].

According to a few LCA study results, WPCs outperformed individual wood-based as well as plastic materials. Nevertheless, the amount of plastic waste recirculated through WPC ingredients is dependent on the classification of plastic waste, the accessibility of recycled plastic, as well as the manufacturing facilities for WPC pallets. The recycling of mono-materials can be completed, however, by using non-pure plastics as well as fibers, of that kind as those found in mixed plastics as well as other WPC products. Material recovery as well as recyclability there at EoL affects the WPC pallet’s circularity. Nearly half of WPC ingredients were created from wood waste and the other half from plastic waste. The environmental impact of this pallet’s production is limited to the manufacturing and delivery of additives, electricity, and diesel because it is made from waste [278]. Circular economies, on the other hand, have been criticized for their zero-burden approach because waste is no longer seen as waste but as raw resources for those and more reasons [48]. As a result, the environmental impacts of WPC pallets might be exacerbated if plastic and wood have been assigned some of the burdens from their own previous life cycles.

WPC products’ ecological implications are primarily caused by their alternate heat sources, energy source, production energy consumption, and weight. A significant part of the WPC product’s life cycle is also played by carbon-neutral wood incineration as well as waste disposal with zero burdens. Several other studies have found that production WPCs can significantly reduce ecological consequences while also efficaciously managing CDW [279,280,281]. It was also concluded from other studies that the reduction of environmental impacts is highly feasible through the manufacturing of WPCs in order to effectively manage CDW.

## 5. Applications of WPCs

Practical applications of WPCs depend on the hydrophilic nature of cellulose, which results in moisture absorption and dispersion problems. Predominant applications of WPCs include construction, aerospace, and automobile industries but they are also applied for packaging, for the preparation of various household articles, furniture, office appliances, and other related items [282,283]. In most of these applications, they are used as structural materials, where the load-carrying capacity of the wood-based reinforcements play a significant role. This is determined by the particle characteristics of the reinforcing wood-based materials and by interfacial adhesion between the wood-based reinforcement and matrix [284].

WPCs are predominantly used in traffic materials, furniture building, construction, and military applications. WPCs are also becoming more significant materials in many other engineering applications. Moreover, WPCs are also used in civil, marine, and automotive engineering applications. Owing to the advancements in wood-based raw materials, their durability and mechanical characteristics during the production of WPCs, a few other outdoor applications were added to the list including utility poles, fences, decks, and building exterior wood works [285,286]. WPCs were also considered to be the most sustainable and quality-rich when compared with other traditional composites and individual wood-based materials. Wood-based flooring is considered to be the highest application volume of WPCs and it encompasses top veneers of laminated flooring, parquet flooring, and solid plank flooring. Majorly, walnut, maple, red oak, and ash are the types of woods used for composite flooring [287]. Patented sports applications of WPCs include laminated skis, club heads in golf courts, hockey sticks, baseball bats, bows, and gun stocks. With regards to musical instruments, WPCs are used for the manufacturing of bagpipe chanters, flute mouthpieces, wind instruments, and stringed instrument finger boards [288]. In order to manufacture scratch-resistant furniture, WPCs along with wood veneers were used. Products include tabletops, desks for writing, clock faces, plaques, and knife handles. Handrails are another class of small volume applications of WPCs. Handrails are commercial applications which are primarily installed in various locations such as malls, department stores, and airports, for their strength improvements, aesthetics, and ease of maintenance [289]. From all the above discussions, it could be stated that WPCs are predominantly used in low and medium density applications at a wider spectrum.

## 6. Conclusions

Wood polymer composites are considered to be a potential and promising material from environmental and economic viewpoints since they involve the use of recycled wood or wood flour. WPCs exhibited better strength and high marketability at a relatively low cost. Properties of WPCs were directly influenced by the processing and post-processing methods. WPCs fabricated with appropriate processing methods had lower contact angles, less color change, better physical and mechanical characteristics, and relatively higher decomposition temperatures. Addition of fillers and lubricants reduced the solid content in WPCs and reduced the viscosity, thus enhancing the bond strength of WPCs. As per most of the studies, WPCs were also manufactured using bio-based adhesives such as starch, tannin, lignin, and soy protein-based adhesives which were considered to be an environmentally friendly alternative for the conventional synthetic adhesives despite their demerits such as high viscosity, low bonding strength, and high hydrophilicity. Analyzing these demerits and overcoming them in a laboratory scale is still a challenging task to many researchers. Yet, the aforesaid bio-based adhesives could reduce the environmental mitigations caused in the form of formaldehyde emissions and formation of volatile organic compounds during the fabrication of WPCs. Life-cycle assessment studies were also carried out on WPCs to assess their environmental effects and from the results, it was found that the WPCs manufactured from bio-adhesives exhibited lesser effects when compared with synthetic resins such as urea-formaldehyde and phenol-formaldehyde. Further research to develop an assessment method for enhancing the durability and long-life of WPCs can be developed based on the conclusions arrived. Overall, it could be stated that the utilization of recycled wood-based materials to manufacture wood-polymer composites is highly viable with regards to eco-friendliness and contributes to the improvement circular economy. Meanwhile, it also saves the usage of virgin materials, thus enhancing the sustainability in production of composite materials.

## Figures and Tables

**Table 1:** Use of coupling agents, additives, and lubricants for manufacturing WPCs.

Sample No.	Wood Reinforcement	Matrix Material	Content of Wood (wt.%)	Coupling Agent	Lubricant and Additives	Ref.
1.	Poplar wood	High density polyethylene (HDPE)	38–58	Modified PE (3%)	Cellulose nanoparticle additives (5%)	[92]
2.	Beech wood	HDPE	20–50	Modified PE (5%)	-	[93]
3.	Pine wood	HDPE	35 & 40	Modified PE (3%)	Commercial lubricant (2–10%)	[94]
4.	Eucalyptus wood	HDPE	55	Modified PE (7%)	Commercial lubricant (2–10%)	[95]
5.	Pine wood	HDPE	40 & 50	Ester (5%)	-	[96]
6.	Maple wood	HDPE	30–60	-	Thermoplastic silicone additive	[97]
7.	Maple-pine wood hybrid	HDPE	40 & 60	-	-	[98]
8.	Maple wood	HDPE	55–60	Modified PE (2%)	Ethylene bis-stearamide additive (1%)	[99]
9.	Maple wood	HDPE	30–40	-	Zinc stearate additive (2%)	[100]
10.	Lignocel wood	Low density PE (LDPE)	10, 75	-	-	[101]
11.	Birch wood	PP	40	-	Struktol additive (1.9–3.12%)	[102]
12.	Lignocel wood	PP	15, 80	-	-	[101]
13.	White fir wood	PP	35	-	Thermoplastic elastomer additives (50%)	[103]
14.	Pine-firewood hybrid	PP	50, 80	-	-	[104]
15.	Wood flour	PP	30	Modified PP (1%)	-	[105]
16.	Wood flour	PP	50	Modified PP (3%)	Thermoplastic silicone additive (1%)	[106]
17.	Bagasse of wood	Acrylonitrile styrene acrylate	50	-	Styrene butadiene rubber additives (15%)	[107]
18.	Poplar wood	Polystyrene	10–50	-	-	[108]
19.	Whitewood flour	Polyvinyl alcohol	30	-	-	[109]
20.	Wood flour	Vegetable oil derived biopolymer	20	-	-	[110]
21.	Poplar wood	Thermoplastic polyurethane	10–40	Polyolefin grafted maleic anhydride, chitosan and diphenyl methyl propane diisocyanate (5%)	-	[111]
22.	Spruce wood	PLA	10–30	-	-	[112]
23.	Pine wood	PP	20–60	Modified PP (4–17%)	-	[113]
24.	Pine wood	PP	30	Modified PP (7–9%)	Struktol additive (1–2%)	[114]
25.	Wood flour	PP	45	Modified PP (5%)	Stearic acid lubricant (3–5%)	[115]
26.	Wood flour	PP	10–45	Modified PP (2%)	-	[116]
27.	Bamboo wood	PP	30–50	Modified PP (3–5%)	-	[117]
28.	Russian fir wood	PP	50–70	-	-	[118]
29.	Poplar wood	PP and HDPE hybrid	70	Maelic anhydride (0.5–2%)	-	[119]
30.	Poplar wood	LDPE	40	Modified PE (4%)	-	[120]
31.	Birch wood	LDPE	-	-	Ethylene propylene diene monomer rubber lubricant (2%)	[121]
32.	Aspen wood	HDPE	-	Grafter wax and PE (4–8%)	-	[122]
33.	Maple wood	HDPE	30 and 40	Silane (3.5%)	Mineral additives (5%)	[123]
34.	Bamboo wood flour	HDPE	10–60	Modified PE (1%)	Paraffin lubricant (7%) and organic halides (1%)	[124]
35.	Maple wood	HDPE	70	-	-	[125]
36.	Spruce wood	HDPE	20–65	Modified PE (3%)	-	[126]
37.	Mangrove wood	HDPE	10–30	-	-	[127]
38.	Eucalyptus wood	HDPE	50	Modified PE (7%)	-	[128]

## Data Availability

Not applicable.
